# QM/MM Study of the Enzymatic Biodegradation Mechanism of Polyethylene Terephthalate

**DOI:** 10.1021/acs.jcim.1c00394

**Published:** 2021-06-04

**Authors:** Sergio Boneta, Kemel Arafet, Vicent Moliner

**Affiliations:** † Institute of Advanced Materials (INAM), Universitat Jaume I, 12071 Castelló, Spain; ‡ Departamento de Bioquímica y Biología Molecular y Celular, Facultad de Ciencias, Instituto de Biocomputación y Física de Sistemas Complejos (BIFI), Universidad de Zaragoza, 50009 Zaragoza, Spain

## Abstract

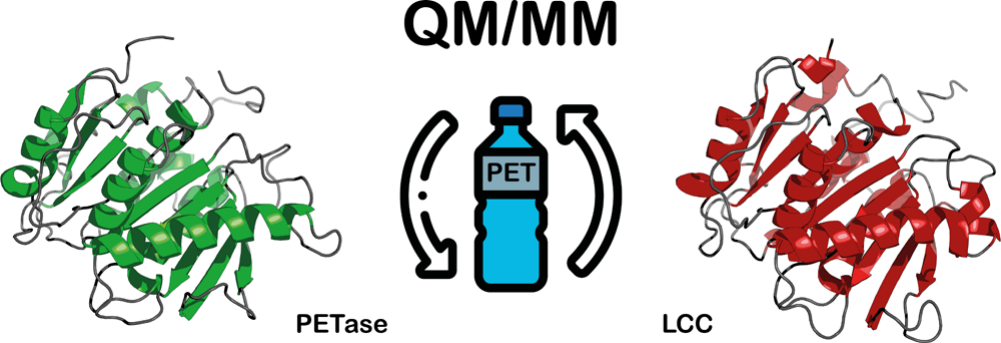

The environmental problems derived from the generalized plastic consumption and disposal could find a friendly solution in enzymatic biodegradation. Recently, two hydrolases from *Ideonella sakaiensis* 201-F6 and the metagenome-derived leaf-branch compost cutinase (LCC), more specially the improved ICCG variant, have revealed degradation activity toward poly ethylene terephthalate (PET). In the present study, the reaction mechanism of this polymer breakage is studied at an atomic level by multiscale QM/MM molecular dynamics simulations, using semiempirical and DFT Hamiltonians to describe the QM region. The obtained free energy surfaces confirmed a characteristic four-step path for both systems, with activation energies in agreement with the experimental observations. Structural analysis of the evolution of the active site along the reaction progress and the study of electrostatic effects generated by the proteins reveal the similarity in the behavior of the active site of these two enzymes. The origin of the apparent better performance of the LCC-ICCG protein over PETase must be due to its capabilities of working at higher temperature and its intrinsic relationship with the crystallinity grade of the polymer. Our results may be useful for the development of more efficient enzymes in the biodegradation of PET.

## Introduction

Management of plastic wastes accumulated in landfills manifests as one of the major challenges pending to be seriously faced in the near future. Only 15% are recycled in Europe^1^ and less than 10% are recycled worldwide.^2^ Poly ethylene terephthalate (PET) accounts for the sixth most-produced polymer and the most commonly manufactured thermoplastic, principally for packaging purposes.^3^ Of the total amount produced, almost 42% was recycled in the EU in 2017.^4,5^ However, its difficulty to be chemically broken into monomers leaves the mechanical reconversion as the principal technique available. Nevertheless, physical properties are inevitably lost after the process, so it may not be suitable for the production of many products (such as the ubiquitous transparent bottles), thus keeping virgin polymer as an irreplaceable source. There is a pressing necessity to find manners to depolymerize PET into its raw materials and to do it in a more environmental-friendly way as with the current combinations of mechanical and chemical processes.^6^

In this context, enzymatic degradation arises as an ideal solution to the problem. A lot of work in this direction has been carried out in the recent years.^7−10^ One of the most recent outstanding discoveries in this field was reported on the mesophilic bacteria *Ideonella sakaiensis* 201-F6, revealing the ability to grow in PET environments, degrading, and using it as a carbon energy source.^11^ The study of this microorganism revealed a synergic behavior of two enzymes, a PET hydrolase (PETase) and a mono(2-hydroxyethyl) terephthalate hydrolase (MHETase). Isolation and characterization of the former protein confirmed its responsibility of the chemical breakage of the plastic polymer into monomers, principally MHET. Then, MHETase is capable of breaking this molecule into ethylene glycol and terephthalate, two compounds that can be metabolically assimilated. PETase is currently the best wild-type protein known that can work for this purpose under mild conditions (40 °C). Consequently, many efforts have been put in trying to improve its efficiency and understanding its behavior.^12−20^ Another interesting enzyme with hydrolytic properties against PET can be found on the metagenome-derived leaf-branch compost cutinase (LCC).^21,22^ This alternative enzyme possesses improved thermophilic properties, capable of working at up to 80 °C. This is a feature with two benefits: higher temperatures facilitate larger turnover numbers because of kinetic principles and can also suppose a crucial factor when trying to address the main body of PET. Polymers can appear in a variable crystallinity grade, and it has been shown that the higher the content of the amorphous phase in the plastic, the higher the enzyme activity. Being closer to the glass transition temperature for PET, established at around 60–80 °C in water,^23^ may provide a higher percentage of the amorphous phase in the plastic and foster the chain mobility between the core and the protein-accessible surface. Recently, a mutation over its sequence (F243I/D238C/S283C/Y127G) has been claimed to obtain a remarkable 98-fold improvement in performance.^24^

**Scheme 1:**
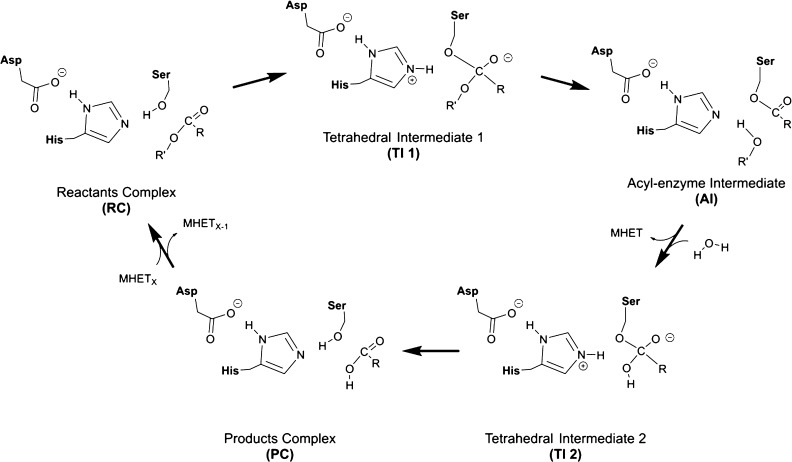
Proposed Mechanism for Hydrolyzation of PET Polymer by PETase and LCC Enzymes The numeration of the displayed residues corresponds to Ser-160/165, Asp-206/210, and His-237/242 for each protein. The polymeric size of the substrate (X) has been explored for 2 on both systems and 3 in PETase.

PETase and LCC can be considered among the most promising candidates available for the PET hydrolysis. Both are structurally similar, with an identity of 49.5% and a α-/β-hydrolase fold with a core of seven α-helices and nine β-strands. They share the same classic nucleophile-histidine-acid catalytic triad, with serine and aspartic acid residues (PETase: S160/H237/D206, LCC: S165/H242/D210).^9^ The classic four-step mechanism proposed for this type of enzymes relies on the acylation of the serine, concomitant with the breaking of the polymer, followed by its hydrolysis assisted by a water molecule of the solvent (Scheme 1).^25^ The attack of the ester bond of the polymer by the serine forms a *tetrahedral intermediate* (TI 1), and then, the first product of the reaction, MHET (or a fragment of the polymer, depending on the length of the initial polymeric chain and the breaking site), is released, and an *acyl-enzyme intermediate* (AI) is formed. The liberation of a molecule of MHET from the active site facilitates the pose of a water molecule that can perform a backwards process. This is the attacking of the new ester bond of the AI to form a secondary *tetrahedral intermediate* (TI 2) that is finally decomposed to release the second product of the reaction: another MHET molecule (or a shorter polymeric chain).

Apart from structural speculation from crystallographic electron density maps, very few pieces of explicit evidence of the mechanism are available in the literature for these two enzymes.^12−16,19,26^ Some computational attempts to elucidate the docking of the polymer to the PETase have already been published,^12,14^ although some have confronted severe experimental disagreements.^27^ Classical molecular dynamics (MD) simulations have also been used to try to determine the influence of the residues on the active site and its flexibility^15,16^ and in the case of the LCC protein to model the accommodation of its substrate.^24^ However, the catalytical mechanism has not yet been theoretically studied for any PET-breaking enzyme. In the case of MHETase, the reaction mechanism of the hydrolysis of a MHET molecule catalyzed by this protein from *I*. *sakaiensis* has been recently explored with QM/MM methods by Knott *et al.*(28) Employing semiempirical force fields (SCC-DFTB), they reported a two-step serine hydrolase mechanism, where both the formation of the acyl-enzyme intermediate (acylation) and its subsequently hydrolysis (deacylation) take place as single steps, without any stable tetrahedral intermediates. According to the authors, the deacylation step arises as the rate-limiting step for the MHET breakage process, with a free energy barrier of 19.8 kcal·mol^–1^ at 30 °C.^28^

This paper focuses on studying the mechanism of action of the PETase enzyme and the LCC-ICCG variant at an atomistic level by means of multiscale QM/MM MD simulations. Independent calculations were carried out using a dimer and a trimer of PET as the substrate. Based on the results, the same mechanism was explored for the LCC-ICCG protein with a dimer. Free energy surfaces (FESs) in terms of potential of the mean force (PMF) have been obtained, thus allowing a detailed description of the mechanism. The results, after a successful comparison with the available experimental data, may be valuable to propose possible mutations, leading to more efficient PET-degrading enzymes.

## Results and Discussion

### Mechanism of PETase/MHET_2_

Our first goal was to describe the mechanism of hydrolysis of PET catalyzed by PETase. For this purpose, a model of this system was prepared with a dimer (MHET_2_) manually docked in the active site of the enzyme, as described in the Computational Methods section. The substrate is broken symmetrically in such a way that two MHET molecules are obtained at the end of the reaction, consistent with the evidence that this is the major product of the enzyme.^11^ To carry out the simulations, a temperature of 313 K was chosen, being the optimal one for the protein effectiveness.^9^

**Figure 1:**
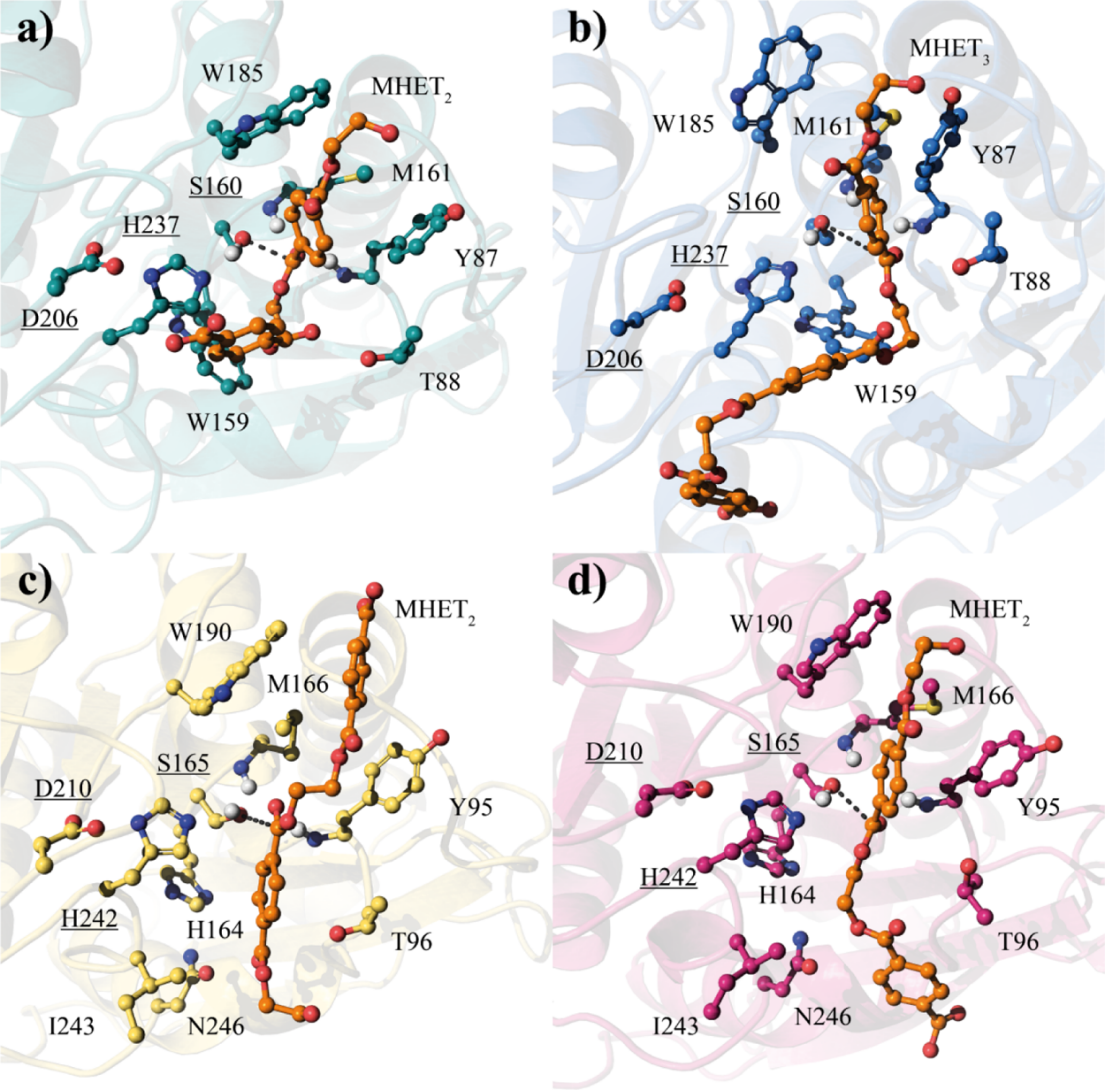
Representation of the different models of the PET molecule (orange) docked in the active site of PETase and LCC. (a) PETase (in green) with HMET_2_; (b) PETase (in blue) with HMET_3_; (c) LCC-ICCG (in yellow) with HMET_2_ in a “flipped” orientation; and (d) LCC-ICCG (in red) with HMET_2_ in the “normal” disposition. The distance defining the nucleophilic attack between the serine and substrate’s oxygen that takes place in the first step is represented as a black dashed line.

An important condition in the setup is regarding the orientation of the substrate in the active site, which determines which ester group will be hydrolyzed. Little experimental information is available, with only a monomer of the plastic (MHET) and an analogous molecule (*p*-nitrophenol) successfully crystalized in the active site.^13^ The consensual disposition for the PETase considers the first ester group after the aromatic ring as the most suitable for the nucleophilic attack.^9^ This scheme allows an un-hindered accessibility to the serine. The aromatic ring of the ligand also correctly disposes to interact with the nearby Tyr-87 and Trp-185, and the substrate fits adequately to the oxyanion hole formed by the backbone nitrogen atoms of Tyr-87 and Met-161. Therefore, this is the disposition that was used in our simulation, as displayed in Figure 1a.

**Figure 2:**
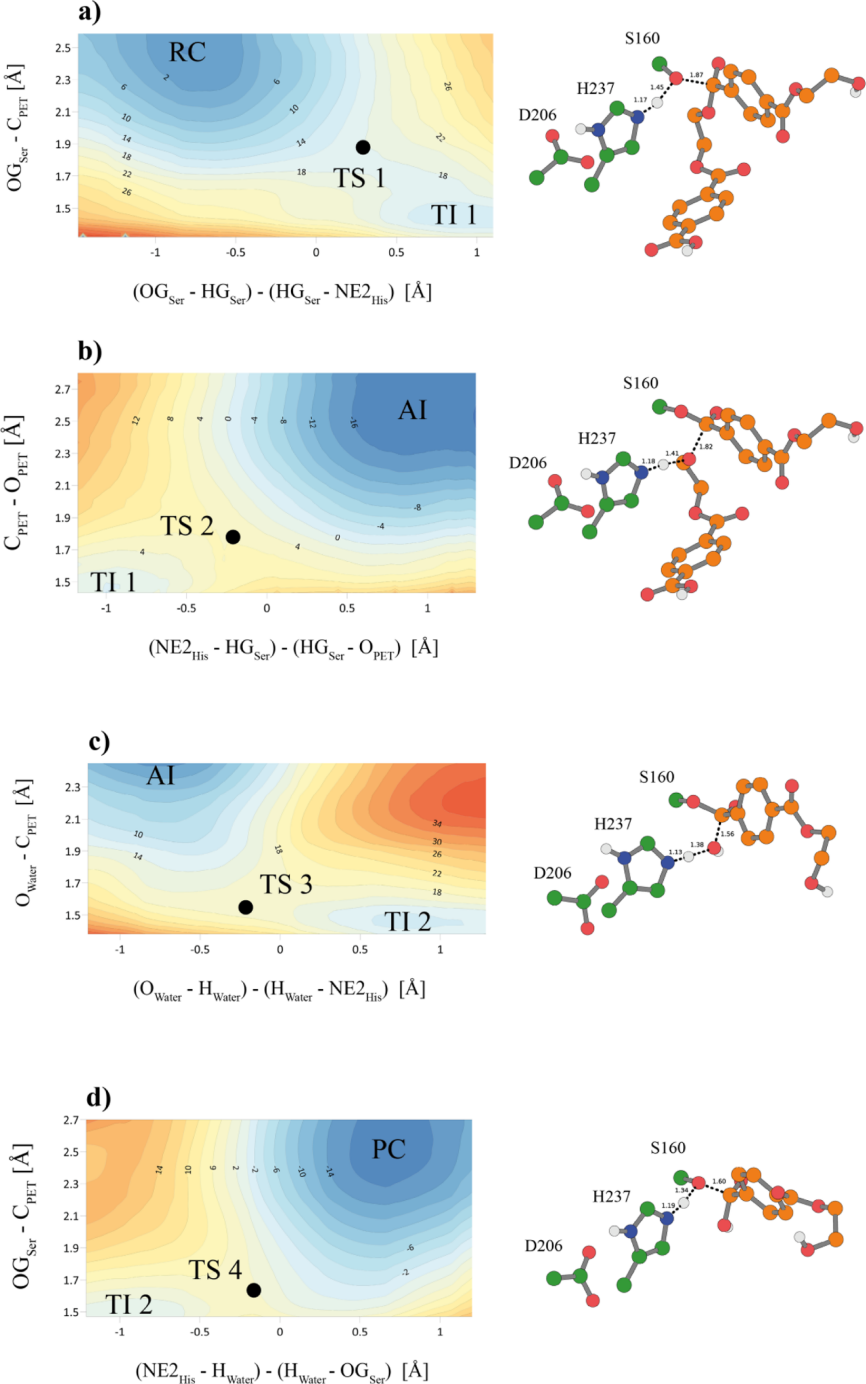
M06-2X/6-31+G(d,p):AM1/MM FESs corresponding to the reaction mechanism of the PETase/MHET_2_ system: nucleophilic attack step (a); acyl-enzyme intermediate formation and release of the first product (b); formation of the second tetrahedral intermediate (c); and formation and release of the second product (d). Energy differences are denoted with contour labels (kcal·mol^–1^). Details of the corresponding QM-atoms of each transition state optimized at the M06-2X/6-31+G(d,p)//MM level are represented with balls and sticks and displayed in the surfaces as black dots.

The M06-2X/6-31+G(d,p):AM1/MM FESs for the whole reaction mechanism are shown in Figure 2. The corresponding PMFs at the AM1/MM level are deposited in the Supporting Information (Figure S6). As can be observed, the surfaces confirm that the reaction mechanism consists of four steps. In the **first step**, the nucleophilic attack of the Ser-160 to the ester’s carbon atom of the polymer occurs in a concerted manner with the proton transfer from Ser-160 to His-237. According to the results, the first reaction proceeds with a free energy barrier of 17.7 kcal·mol^–1^, leading to the formation of the tetrahedral intermediate 1 (TI 1), 14.4 kcal·mol^–1^ less stable than the reactants.

The **second step** leads to a stable *acetyl-enzyme intermediate* (AI) through the breaking of the ester bond of the polymer concomitant with the serine–substrate bond formation. The exergonic reaction liberates 17.7 kcal·mol^–1^ and needs 5.9 kcal·mol^–1^ to reach the transition state, TS 2, in a concerted fashion. After the reaction, the leaving group (a molecule of MHET) is removed from the active site, and the system is re-equilibrated with unconstrained AM1/MM MD simulations (see the Computational Methods section for details).

For the **third step**, a solvent water molecule accesses the active site from the same side as the catalytic triad to allow keeping the interaction between the carbonyl oxygen of the acyl-enzyme and the oxyanion hole provided by the Tyr-87 and Met-161 backbone in the opposite side. The water oxygen bounds to the carbonyl carbon of the remaining substrate that is attached to Ser-160. Concertedly, water’s proton is transferred to the His-237 nitrogen atom. Thus, a second tetrahedral intermediate (TI 2), similar to the one obtained after the first step, is reached. According to the FES of the reaction, an activation energy of 16.9 kcal·mol^–1^ is required.

Finally, in the **fourth step**, the second product is released in the form of another MHET molecule, and the enzyme returns to its initial state, ready for another catalytic cycle. This last concerted step, requiring an energy of 5.0 kcal·mol^–1^, is clearly exergonic: 17.1 kcal·mol^–1^.

In an effort to validate the obtained results with a high level of theory, the four transition states were optimized at the M06-2X/6-31+G(d,p)/MM level. The resulting structures, characterized as transition-state structures with their corresponding single-negative frequencies (−539.2, −539.8, −431.8, and −420.4 cm^–1^ for TS 1, TS 2, TS 3, and TS 4, respectively) were found to be close to those selected from analysis of the quadratic region of the corrected M06-2X/6-31+G(d,p):AM1/MM FESs.

### Mechanism of PETase/MHET_3_

**Figure 3:**
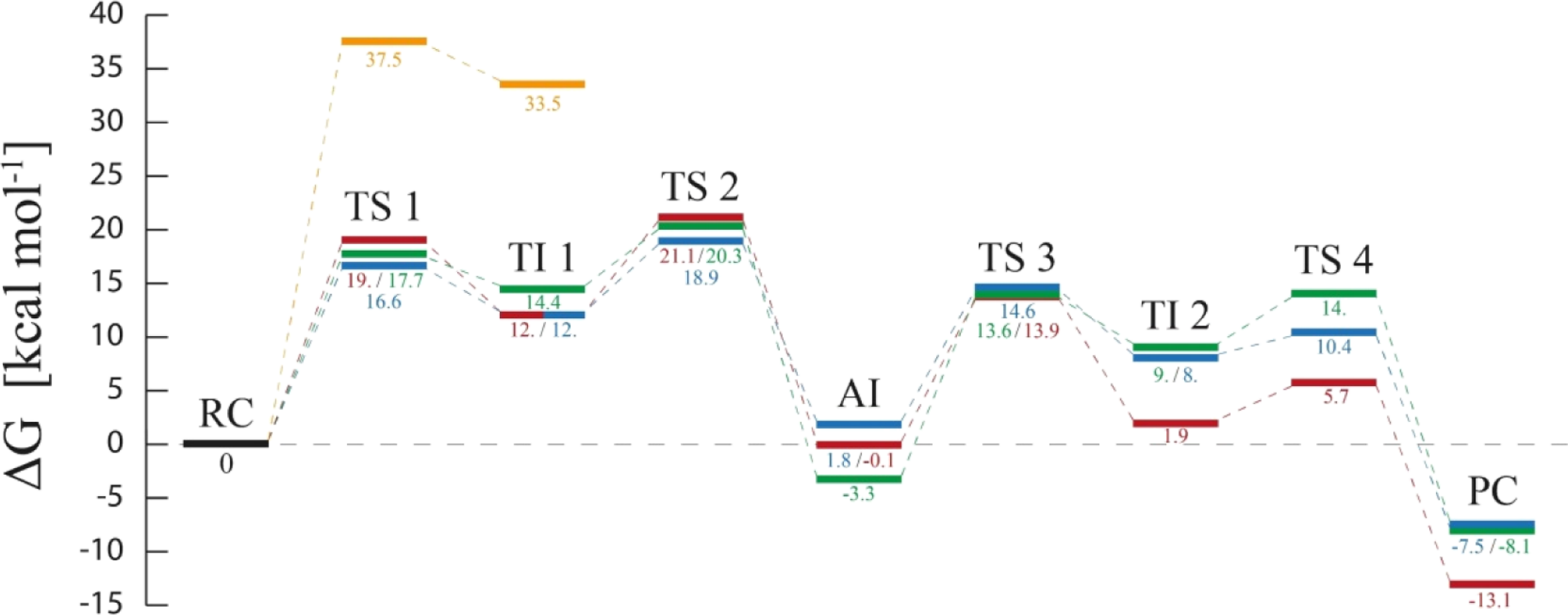
Free energy profile of the PET polymer breakage reaction derived from FESs computed at the M06-2X/6-31+G(d,p):AM1/MM level of theory. Each color represents an independently studied system: PETase/MHET_2_ (green), PETase/MHET_3_ (blue), and LCC-ICCG/MHET_2_ in a “flipped” disposition (yellow) and LCC-ICCG/MHET_2_ in the “normal” orientation (red).

Despite the limited size of a PET dimer as the substrate model in comparison with a complete polymer structure, it must be large enough to correctly mimic the reactive behavior of the active site. Nevertheless, in order to explore the possible polymer chain length dependency of our results, a second model of the PETase enzyme was prepared with a trimer of PET (MHET_3_) as the substrate. The increased size of the substrate goes a step forward in the modeling of a large polymeric substrate and, according to the pose of the molecule in the active site, the substrate–protein interactions remain equivalent. Moreover, the resulting FESs (see Figures S3 and S7 of the Supporting Information) are equivalent to those reported in Figure 2 for the dimer at the same level of theory, showing energy differences below 2 kcal·mol^–1^. Comparison of the free energy profile for reaction in the PETase/MHET_2_ and PETase/MHET_3_ models is shown in Figure 3.

### Mechanism of LCC-ICCG/MHET_2_

As mentioned in the Introduction section, recent publications have moved the point of interest of the enzymatic degradation of the PET polymer to the leaf-branch compost cutinase (LCC). Being a more thermophilic candidate for the same hydrolysis reaction, after some mutations, it has been claimed to have better efficiency while working at higher temperatures.^10,24^ For a better comprehension of this protein, the best reported variant, denoted as ICCG (F243I/D238C/S283C/Y127G), was explored in the present study following the same computational strategy as the PETase to describe its specific mechanism and to elucidate the reason of its improved performance. As shown in Figure 1, both active sites are nearly identical, with the same catalytic triad and surrounding residues. Considering the substrate length independency obtained with PETase, just a dimer molecule (MHET_2_) was used as the substrate for the calculations, as it saves a considerable amount of computational resources.

The dimer substrate was *a priori* posed in the active site of LCC-ICCG as in the case of the PETase “normal” orientation. Nevertheless, Tournier et al. stated that a “flipped” disposition may be more convenient for the LCC enzyme, based on classical docking and short MD simulations.^24^ With this orientation, the second ester group should be the one to be broken. In order to clarify this situation, both dispositions were independently prepared and studied for the LCC-ICCG enzyme (Figure 1c,d). The temperature of the simulations, set to 313 K, may not take full advantage of LCC possibilities, capable of working at up to 72 °C,^24^ but ensures a fair comparison with the results derived from the study of PETase.

Due to the high similarities of the active sites between LCC-ICCG and PETase, the explored molecular mechanism was the same as presented in Scheme 1. In the **first step**, the same kind of concerted mechanism was found for both possible dispositions of the substrate (FESs at the M06-2X:AM1/MM level are shown in Figures S4 and S5a of the Supporting Information, while those at the AM1/MM level are in Figure S8). Nevertheless, a remarkable difference in energy barriers arises. While the activation free energy of this first step with the “normal” orientation was 19.0 kcal·mol^–1^, similarly to the PETase, the barrier obtained with the “flipped” orientations was significantly higher, 37.5 kcal·mol^–1^, far beyond reasonable values of enzyme catalysis under normal conditions. Therefore, the remaining steps of the mechanism were only explored with the “normal” orientation.

The **second step** presents an energy barrier of 9.0 kcal·mol^–1^ to reach an *acetyl-enzyme intermediate* (AI) with an energy of 12.1 kcal·mol^–1^, lower than that of the preceding tetrahedral intermediate. In the **third step**, the hydrolysis of the AI to render the *tetrahedral intermediate 2* appears to be a slightly endergonic process, 12.0 kcal·mol^–1^, requiring an activation free energy of 14.0 kcal·mol^–1^. The **fourth step** appears to be also exergonic, 15.0 kcal·mol^–1^, with a low barrier of 3.8 kcal·mol^–1^.

### PETase/MHET_2_*Versus* PETase/MHET_3_*Versus* LCC-ICCG/MHET_2_

All three studied systems follow the same mechanism to degrade PET, with minor variations. To directly carry out a direct comparative analysis, the three **free energy** profiles are presented together in Figure 3. The rate-limiting step for the global process, corresponding to the TS 2 in all three systems, requires an activation free energy of 20.3, 18.9, and 21.1 kcal·mol^–1^ for the PETase/MHET_2_, PETase/MHET_3_, and LCC-ICCG/MHET_2_, respectively. The following deacylation process needs a slightly lower activation energy, 14.0, 14.6, and 13.9 kcal·mol^–1^, respectively. The agreement is highly remarkable between the calculated barriers for the PETase with MHET_2_ and MHET_3_ substrates, thus confirming the absence of any dependency on the length of the polymer chain and relying exclusively on the inner radius of the active site. Regarding the LCC-ICCG, as commented above, the “flipped” disposition (yellow line in Figure 3) clearly can be discarded as a viable reactive complex because of the huge energy barrier of the first step, in contrast to the “normal” orientation that is in the range of PETase. Thus, the apparent performance difference between enzymes cannot be attributed to mechanism differences.

Comparison of the mechanism described herein with the one recently proposed by Knott *et al.* on the breaking of a MHET molecule by MHETase^28^ reveals significant discrepancies. According to their results, a two-step pathway for the full reaction, without localization of intermediates in the acylation or deacylation steps, is proposed based on free energy surfaces at the semiempirical SCC-DFTB level of theory. In their case, the rate-limiting step would correspond to the deacylation, with a free energy barrier of 19.8 kcal·mol^–1^, while they predict an acylation free energy barrier of 13.9 kcal/mol. It is important to point out that in contrast with our study, the authors employed just two distances as reaction coordinates, which can be the origin of certain bias. The authors also performed experimental activity assays to determine MHET turnover rates (*k*_cat_) for MHETase of 27.6 ± 2.6 s^–1^ that would correspond to an energy barrier of 15.6 kcal·mol^–1^.

Structural analysis of the representative structures of the key states involved in the reaction (see Tables S1–3 of the Supporting Information) with the three models studied herein reveals minor differences. The OG_Ser_–C_PET_ distance in the *reactant complex* of the PETase-MHET_2_ is shorter (2.50 ± 0.03 Å) than the corresponding distance in the case of the larger MHET_3_ substrate (3.05 ± 0.04 Å) and the one measured in the LCC-ICCG model (3.20 ± 0.04 Å). This feature could be related with a better docking of the ligand to the active hollow. Nevertheless, while the highest activation energy barrier of the first TS 1 corresponds to the LCC-ICCG system, in agreement with the largest distance between the two atoms to be bonded in this step, the difference between the dimer and trimer mode of the PET model does not appear to be significant. On the other hand, the OG_Ser_–C_PET_ distance in the “flipped” disposition of the complex with MHET_2_ in the active site of LCC-ICCG is dramatically larger (3.7 ± 0.05 Å). This large distance appears to be associated to the requirements to accommodate the substrate in the reactant groove that, in turn, provokes the stabilization of a less-reactive conformation of the Michaelis complex, by comparison with the case of the “normal” orientation. As observed in Figure 3, the resulting activation energy of the first step in the LCC-ICCG/MHET_2_ in a “flipped” disposition (yellow line) is dramatically higher, as discussed above. Thus, these geometrical differences in a distance that defines the reaction coordinate of the first step have an expected effect on the resulting free energy barrier.

Another discrepancy between the results obtained with the different models is found in the timing of the events that define the transition state of the first step for PETase. While the MHET_3_ substrate falls in the same position as the LCC-ICCG, indicating a former stretching of the OG_Ser_–C_PET_ bond until about 1.7 Å before proton transfer to the histidine, the dimer of PETase proceeds in the other way around. However, this fact is not reflected in the barrier of the step or in any other derived property. Another relevant difference is the final distance between water’s oxygen and hydrogen after the split of the molecule in the third step. The 2.37 Å distance reached in the LCC-ICCG/MHET_2_ contrasts with the shorter 1.86 and 1.92 Å of the PETase/MHET_2_ and PETase/MHET_3_, respectively. This reflects that the *tetrahedral intermediate 2* reaches a better accommodation at the protein groove in the LCC-ICCG model, lowering the intermediate’s free energy by around 6 kcal·mol^–1^ in comparison with its PETase counterparts. This over-stabilization of the intermediate structure affects the energy of the next step, overall identical among all systems but arriving to a *product complex* with a lower net free energy in the case of LCC-ICCG than its equivalents. The difference observed between the final states is virtually the same as in the TI 2, 6 kcal·mol^–1^.

**Figure 4:**
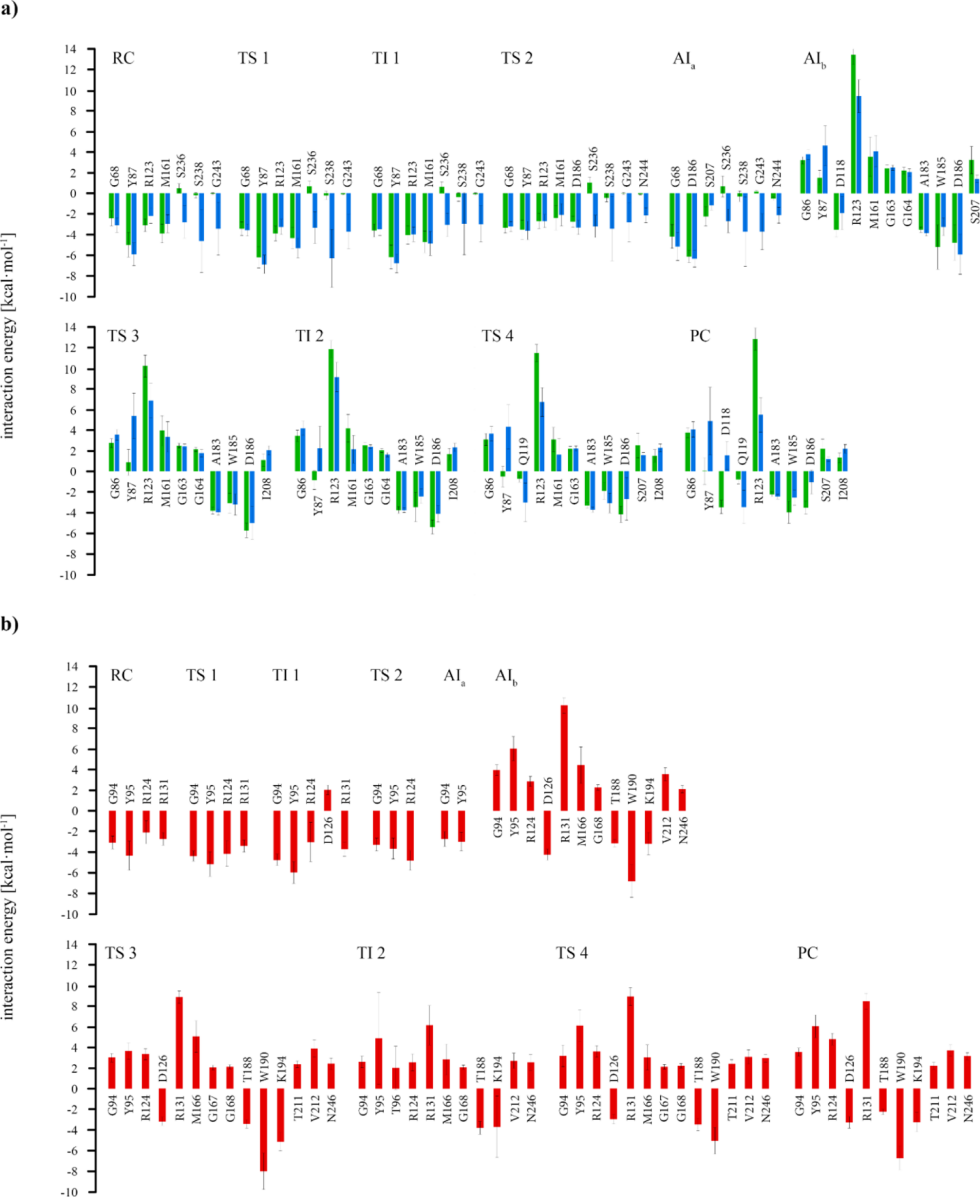
Main electrostatic interaction energies (larger than 2 kcal·mol^–1^, in an absolute value) between the protein and the substrate for all the intermediates and transition structures of the three systems: (A) PETase/MHET_2_ (green) and PETase/MHET_3_ (blue) and (B) LCC-ICCG/MHET_3_ (red). Up to the AI_a_, the leaving group is included in the calculation, but it is removed after AI_b_. The catalytic triad is not included.

The calculation of **electrostatic interaction energies** between the substrate and the protein, presented by the residue in Figure 4, allows us to get an insight into the role of every amino acid in each stage of the reaction. The favorable contribution of the oxyanion hole of Tyr-87/95 is present in every system, but only in the PETase models, the Met-161/166 has a considerable stabilization effect. The stabilizing effects of Ser-236, Ser-238, Gly-243, and Asn-244 in the PETase/MHET_3_, not observed in the dimer case, reveal the cavity of the protein occupied by the extra monomer. No dramatic variations are displayed between reactants/products and transitions states, but the scenario is radically different after the removal of the leaving group and the inclusion of the water molecule for the hydrolysis. This is reflected by the fact that most of the stabilizing residues measured in the first two steps present an unfavorable interaction with the substrate once the AI is formed. Also, new influential residues emerge, being of special relevance, Ala-183, Trp-185, and Asp-186 for PETase and Thr-188, Trp-190, and Lys-194 for LCC-ICCG. The case of Arg-123/131 is another example of residues drastically changing their influence for the hydrolysis. It presents very high positive values of interaction, only lightly contented for the TI 2 in LCC-ICCG, reinforcing the theory of this over-stabilized intermediate.

### Experimental Evidence

In order to validate our computational results, a comparison against experimental data available in the literature on these systems can be made. A reliable way to do so is to compare our computed activation free energies of the rate-limiting step with experimentally determined turnover numbers. Nevertheless, due to the polymeric nature of the substrate and a variable crystallinity grade, this is not a simple task and not many data are available, with inherent discrepancies between published results (Table 1). Originally, the PETase enzyme was characterized with a turnover number of around 0.7 s^–1^ for BHET molecules at 30 °C.^11^ Chen *et al.*(29) reported similar rates of 0.5 s^–1^ for the same substrate and conditions and measured its rate with a PET film of 45% crystallinity at 40 °C with a much slower result of 10^–4^ s^–1^. A remarkable difference can be seen between polymeric and molecular models. The comparison with rates for different molecules, such as pNP variants, also evidences a dependency on the nature of the substrate.^11,29,30^ Overall, it can be concluded that our described mechanism with PETase exhibits a free energy barrier that fits in the range of the experimental data. Unfortunately, no *k*_cat_ values are provided for the LCC-ICCG enzyme; only the non-mutated form has been measured with pNP, reporting similar results.^11,22^

**Table 1:** Experimental Turnover Rates (s^–1^) Available in the Literature for Non-modified PETase and LCC Enzymes, Temperature of Measurement (°C), and Estimated Free Energy of Activation (kcal·mol^–1^) According to Transition State Theory^31^^a^

enzyme	substrate	*T*	*k*_cat_	Δ*G*	ref
PETase	BHET	30	0.7*	18.0	(11)
	BHET	30	0.2*	18.7	(29)
	pNP	30	10*	16.4	(11)
	pNP	30	27.0	15.8	(30)
	pNP	30	1–4*	16.9–17.7	(29)
	PET film	40	0.0001*	24.1	(29)
LCC	BHET	30	0.2*	18.7	(11)
	pNP	30	50–90*	15.0–15.4	(11)
	pNP	30	232	14.5	(22)
	pNP	50	343	15.2	(22)
	pNP	70	213	16.5	(22)

a Marked data (*) are approximated from the original plots. Substrates: BHET, mono(2-hydroxyethyl) terephthalate; pNP, *para*-nitrophenyl acetate/butyrate/caproate/caprylate.

Several PETase studies have explored the effects of mutations on the activity of the enzyme toward depolymerization of PET, of which the most significant are collected by Taniguchi *et al.*(9) Although none of the new proteins resemble the improvement reached by LCC-ICCG compared with its original sequence, Ma *et al.*(30) mutates the residue Ile-208 to phenylalanine, reporting an activity increase by 2.5-fold, indicating a better hydrophobic interaction with the substrate. This is in correspondence with our computational results, where an unfavorable electrostatic interaction is observed between this residue and both MHET_2_ and MHET_3_ in the deacylation process (see Figure 4). It is also interesting how, according to several authors,^13,16^ the activity of the enzyme decreases after mutating Tyr-87, a key residue in the inner active site with favorable influence, into alanine. Nevertheless, others state an increase in the activity.^19^ Overall, the difficulty of evaluating kinetic data derived from mutants of different groups because of the different experimental setups, conditions, and intrinsic sensibility of the measurement has to be remarked.

## Conclusions

In the present work, the breakage mechanism of the PET polymer by the two main enzymes showing certain activity, PETse and LCC-ICCG, has been studied. Three independent systems have been employed: PETase/MHET_2_ and PETase/MHET_3_, to test whether the reactivity depends on the length of the polymer chain, and LCC-ICCG/MHET_2_, to compare the proficiency of both enzymes that work in different ranges of temperatures. A reaction pathway, involving formation of an acyl-enzyme intermediate, acylation stage, and its subsequently released deacylation stage, has been investigated by generating free energy surfaces in terms of potential of the mean force (PMFs) with QM/MM potentials. The QM sub-set of atoms was described at the semiempirical (AM1) and DFT (M06-2X) level of theory. Two possible dispositions of the substrate in the active site have been explored in the case of LCC-ICCG, finding one to be clearly favored over the other. The resulting free energy landscape reveals that both the acylation and the deacylation take place in a stepwise manner in all the three models. In all cases, the rate-limiting step was found to be the second step of the acylation process, with activation free energies of 20.3, 18.9, and 21.1 kcal·mol^–1^, respectively. These results fit in the range of the available experimental data. Structural analysis of the evolution of the active site along the reaction progress and the study of the electrostatic interactions between the substrate and the protein decomposed by residues confirm the similarity in the behavior of the active site of these two enzymes capable of degrading PET in a significantly different range of temperatures. According to our results, the origin of the apparent better performance of LCC protein over PETase cannot be in mechanistic differences of the chemical step but in its capabilities of working at higher temperature and its intrinsic relationship with the crystallinity grade of the polymer. Our results may be useful for the development of more efficient enzymes in the biodegradation of PET, with future applications to address the environmental problems derived from the generalized plastic consumption and disposal.

## Computational Methods

### Preparation of the Systems

The PETase molecular model was built upon the highest-resolved X-ray crystal structure (0.92 Å) of the apoenzyme available in the Protein Data Bank, with code 6EQE.^12^ The LCC model was constructed from the crystallized wild-type structure with PDB code 4EB0(22) and mutated to produce the ICCG variant (F243I/D238C/S283C/Y127G). The proteins were prepared with Maestro software;^32^ disulfide bonds were built and hydrogen atoms present already were deleted and added corresponding to a protonation state of pH 7 with PROPKA.^33^ Before the inclusion of the plastic ligand, both molecular models were relaxed under unconstrained classical MD with GROMACS 2018.4.^34^ For this purpose, explicit solvation consisting of 8560 and 8486 water molecules was added together with 6 Cl^–^ counterions in a rhombic dodecahedron-shaped box. The systems were then statically minimized with the steepest descent and conjugate gradient to avoid clashes. Random velocities were generated at 313 K, and 500 ps of protein-constrained NVT and NPT was run as equilibration. Finally, 50 ns of unconstrained production was carried out under an NPT ensemble with a time step of 1 fs and using AMBER ff03^35^ with TIP3P for water molecules (see Figure S1 of the Supporting Information).

Confirming the equilibration of the systems based on the RMSD of the backbone, the plastic polymer was manually added based on the position of the HEMT molecule co-crystalized in the PDB with code 5XH3.^13^ A similar classic MD procedure as described before was performed to equilibrate the ligand–enzyme models during a long simulation of 200 ns (Figure S2), granting a necessary organization of the active site that, as previously stressed by Smith *et al.*, is required in multi-step enzyme-catalyzed reactions.^36^ The polymers were described with GAFF^37^ parameters assigned with Antechamber^38^ using the RESP atomic partial charges calculated at the HF/6-31G(d) level of theory with Gaussian.^39^

### QM/MM Calculations

**Figure 5:**
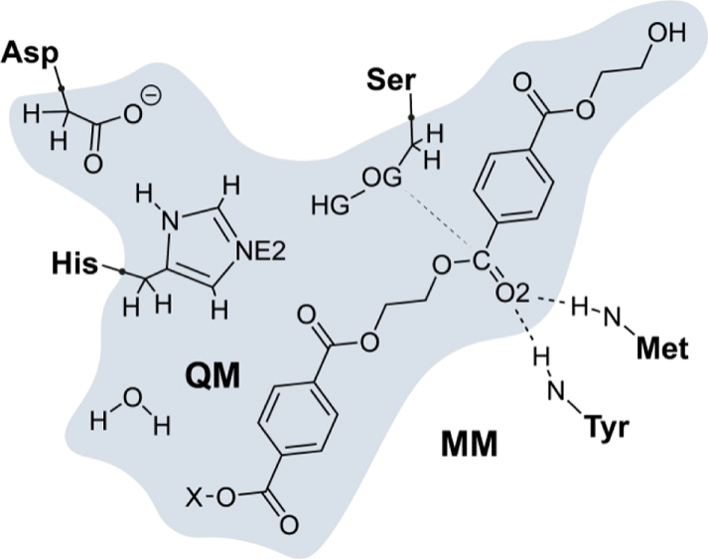
Schematic representation of the active site of PETase and LCC. The subset of atoms in the shaded area is included in the QM region. Displayed residues correspond to the catalytic triad (PETase: S160/H237/D206, LCC-ICCG: S165/H242/D210) and the oxyanion hole (PETase: M161/Y87, LCC-ICCG: M166/Y95). The X substituent in the polymeric substrate can either be a hydrogen atom to form MHET_2_ or another monomer to form MHET_3_. Hydrogen-link atoms are placed in the frontier bonds, represented as black dots.

Favorable (reactive) structures, based on the minimization of the OG_Ser_–C_PET_ distance, were chosen after the MD simulations as starting points for QM/MM calculations with the fDYNAMO library.^40^ The quantum-treated region was confirmed by the polymeric ligand, a water molecule when necessary and key atoms of the catalytic triad (Ser/His/Asp), as depicted in Figure 5. The rest of the protein was described by the OPLS-AA^41^ force field and TIP3P for the solvent.^42^ All atoms farther than 20 Å from the catalytic serine’s oxygen were kept frozen.

A three-layer scheme of calculations was used to quantitatively describe the reaction mechanism. First, the bidimensional potential energy surface (PES) of every step was explored using the AM1^43^ semiempirical Hamiltonian to describe the QM subset of atoms to optimize every structure while being restrained by a harmonic umbrella potential of 3500 kJ·mol^–1^·Å^2^. It was characterized by the localization of structures as minima or transition states if identified as saddle points with AM1/MM. Once completed, free energy surfaces (FESs) in terms of the PMF associated with the selected coordinates were generated by umbrella sampling, consisting AM1/MM MD simulations of 10 ps of equilibration and 20 ps of production with a time step of 1 fs. A harmonic force of 2500 kJ·mol^–1^·Å^2^ was employed, and the weighted histogram analysis method (WHAM)^44^ was used to integrate the data. With this technique, the expected associated error is usually lower than 1 kcal·mol^–1^.^45^ On top of that, a last layer of a high level of theory is employed in an interpolation correction way over the semiempirical results, a methodology successfully tested previously in our laboratory.^46^ The M06-2X functional^47^ with the 6-31+G(d,p) basis set was used, employing Gaussian^39^ software together with the fDYNAMO library.^40^ Selected transition states were also located, employing directly DFT/MM (its coordinates are deposited in Table S4 of the Supporting Information). Averaged geometries were obtained from longer (100 ps) simulations of the structures of interest. The interaction energy of each residue of the protein with the ligand was computed along these trajectories.

## Supporting Information Available

RMSDs for the backbone of all the systems during classical MD simulations with and without a ligand, FESs computed at M06-2X/6-31+G(d,p):AM1/MM and AM1/MM for every step of the reaction and model studied, and key averaged interatomic distances and electrostatic interaction energies for all the residues in every catalytic step and coordinates of the QM atoms for the four transition states located at M06-2X/6-31+G(d,p)/MM (PDF)

## Notes

GROMACS 2018.4 can be freely obtained from www.gromacs.orgAntechamber, part of AmberTools20 that can be obtained from ambermd.org/AmberTools.php. Gaussian 09 D01 can be purchased from gaussian.com. fDYNAMO v2.2 can be freely downloaded from www.pdynamo.org/downloads.
