# Supplementary material for: QM/MM Study of the Enzymatic Biodegradation Mechanism of Polyethylene Terephthalate
Source: J Chem Inf Model. 2021 Jun 4;61(6):3041–51. doi: 10.1021/acs.jcim.1c00394 (PMC8576761; doi:10.1021/acs.jcim.1c00394)
Supplement: Supplementary file 1 — ci1c00394_si_001.pdf [file ci1c00394_si_001.pdf]

Supporting Information

QM/MM Study of the Enzymatic Biodegradation Mechanism of Polyethylene Terephthalate

Sergio Boneta,[1,2] Kemel Arafet,[1] Vicent Moliner[1]

1. Institute of Advanced Materials (INAM), Universitat Jaume I, 12071 Castelló, Spain

2. Departamento de Bioquímica y Biología Molecular y Celular, Facultad de Ciencias, Instituto de Biocomputación y Física de Sistemas Complejos (BIFI), Universidad de Zaragoza, 50009 Zaragoza, Spain

AUTHOR INFORMATION

Corresponding Authors:

*K.A.: e-mail, arafet@uji.es; tel, +34964728069.

*V.M.: e-mail, moliner@uji.es; tel, +34964728084.

ORCID

Sergio Boneta: 0000-0002-7668-7852

Kemel Arafet: 0000-0002-0569-7332

Vicent Moliner: 0000-0002-3665-3391

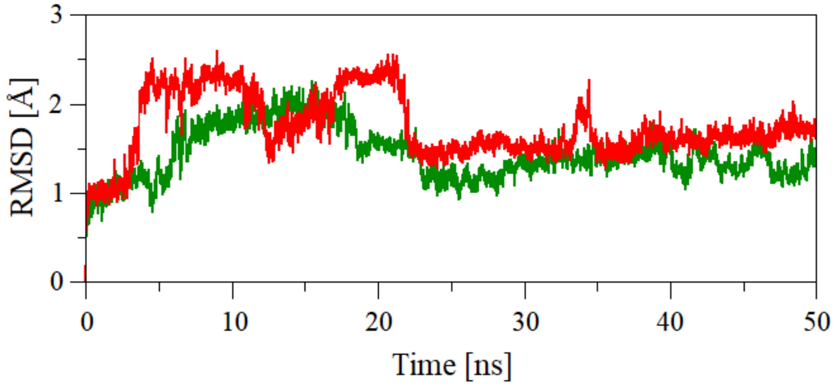

Figure S1. Root mean square deviation (RMSD) of the backbone atoms of PETase (green) and LCC-ICCG (red) during classical-MD simulations of equilibration without ligand.

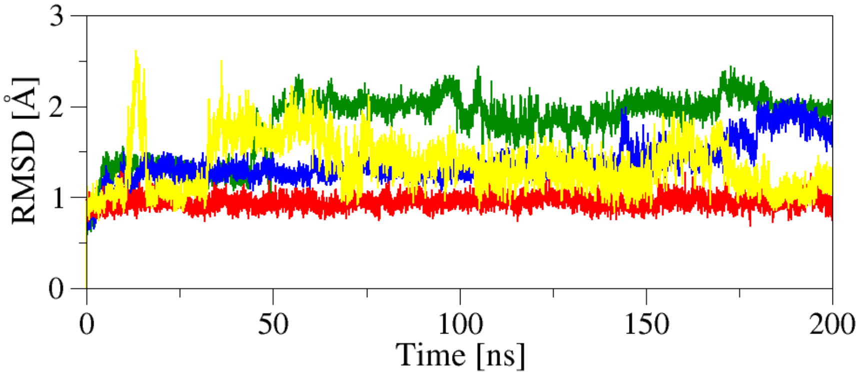

Figure S2. Root mean square deviation (RMSD) of the backbone atoms during classical-MD simulations of equilibration with the substrate in the active site. PETase:$MHET_2$ (green); PETase:$MHET_3$ (blue); LCC-ICCG:$MHET_2$ in "flipped" orientation (yellow) and LCC-ICCG:$MHET_2$ in "normal" disposition (red).

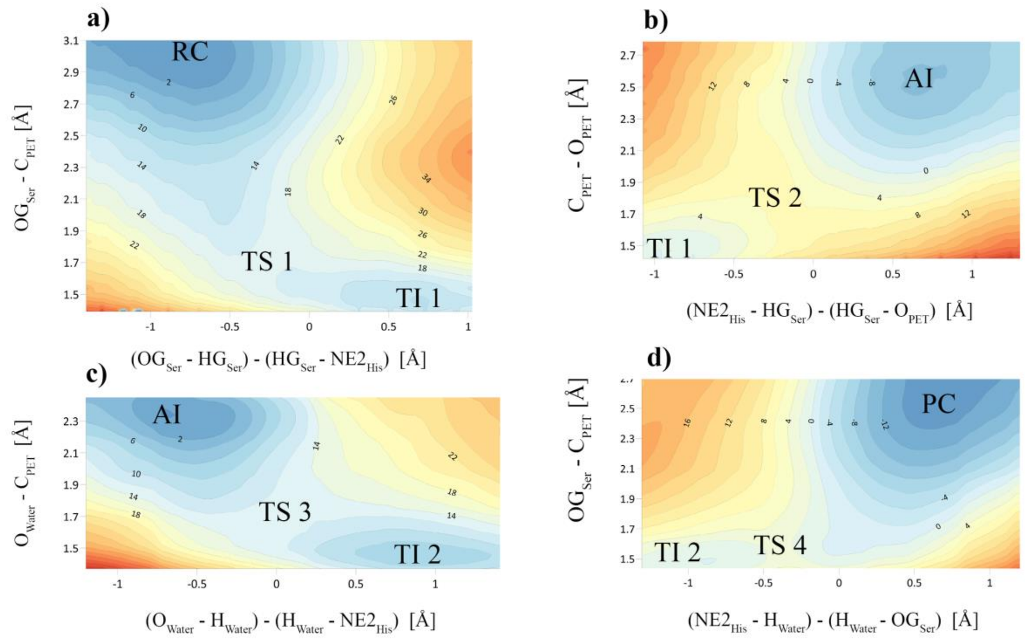

Figure S3. M06-2X/6-31+G(d,p):AM1/MM FESs (kcal·mol^{-1}) corresponding to the reaction mechanism of PETase:MHET$_3$ system: nucleophilic attack step (a), split of the substrate (b), formation of the TI 2 intermediate (c), and formation of the product (d).

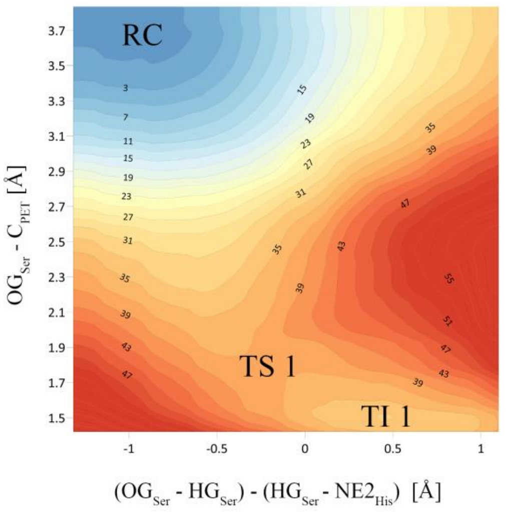

Figure S4. M06-2X/6-31+G(d,p):AM1/MM FESs (kcal·mol^{-1}) corresponding to the reaction mechanism of the initial nucleophilic attack in the LCC-ICCG:MHET$_2$ system with a "flipped" disposition of the substrate.

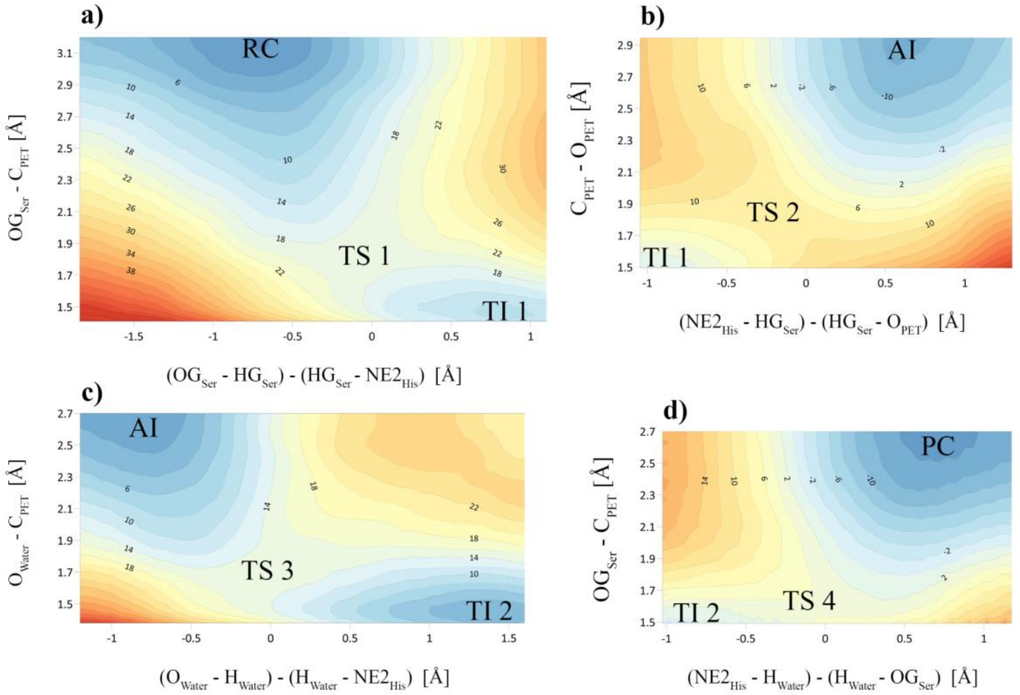

Figure S5. M06-2X/6-31+G(d,p):AM1/MM FESs (kcal·mol^{-1}) corresponding to the reaction mechanism of LCC-ICCG:$MHET_2$: nucleophilic attack step (a), split of the substrate (b), formation of the TI 2 intermediate (c), and formation of the product (d).

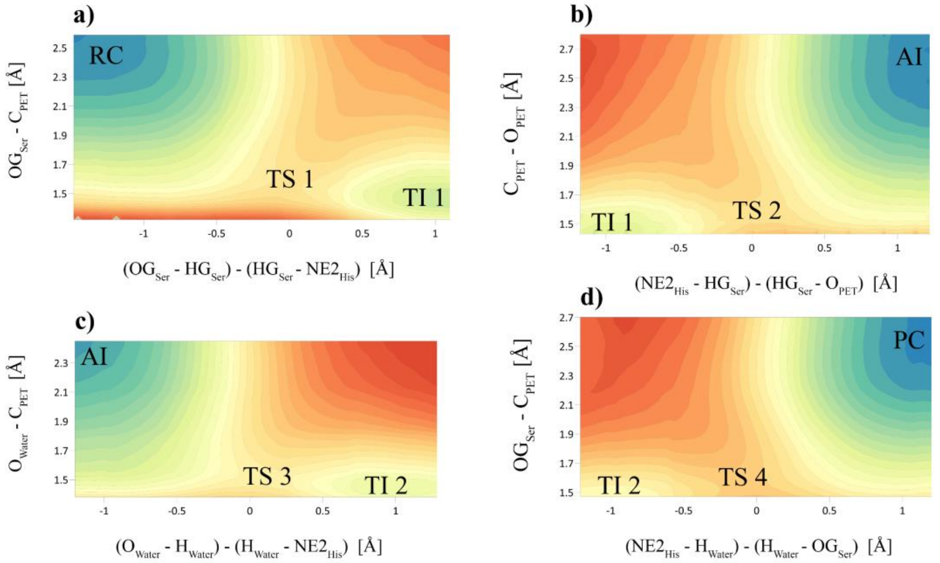

Figure S6. AM1/MM PMFs corresponding to the reaction mechanism of PETase:$MHET_2$: nucleophilic attack step (a), split of the substrate (b), formation of the TI 2 intermediate (c), and formation of the product (d).

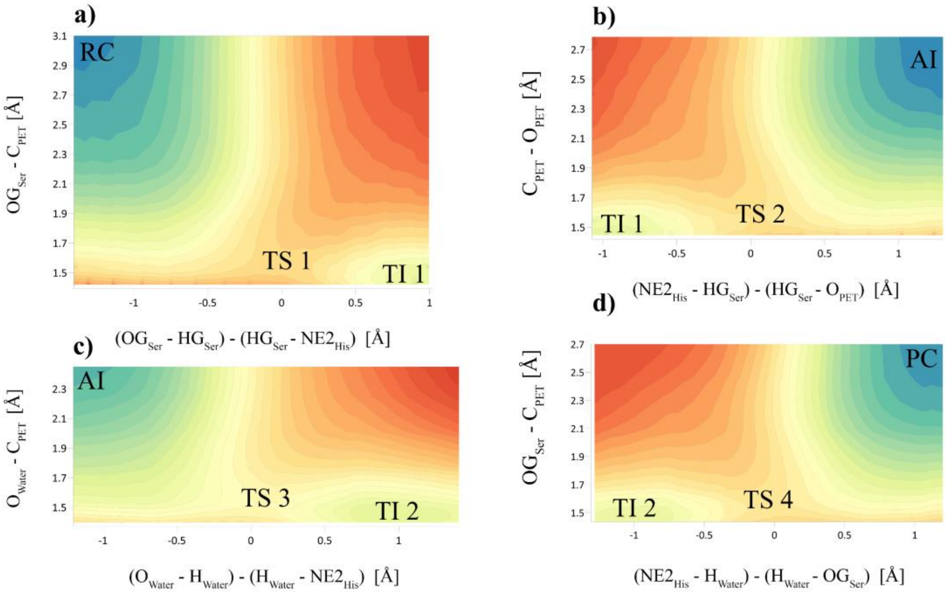

Figure S7. AM1/MM PMFs corresponding to the reaction mechanism of PETase:MHET$_3$: nucleophilic attack step (a), split of the substrate (b), formation of the TI 2 intermediate (c), and formation of the product (d).

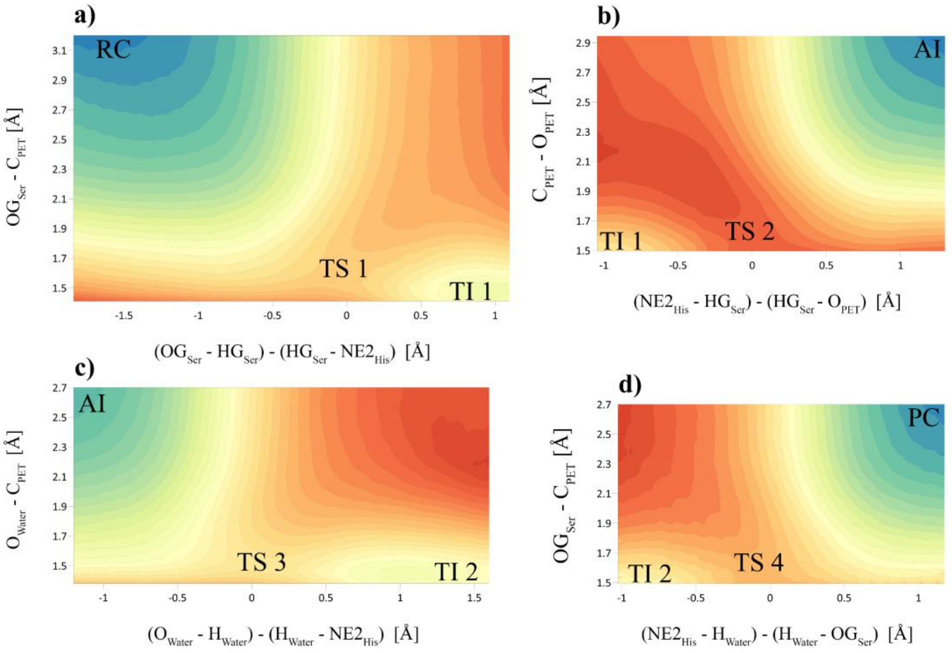

Figure S8. AM1/MM PMFs corresponding to the reaction mechanism of LCC-ICCG:MHET$_3$: nucleophilic attack step (a), split of the substrate (b), formation of the TI 2 intermediate (c), and formation of the product (d).

Table S1. Averaged distances (Å) between key atoms during 100ps AM1/MM MD simulation for different structures along the reaction mechanism explored for the PETase:$MHET_2$ model.

	RC	TS 1	TI 1	TS 2	AI_a
OG_{S160} - C_{PET}	2.50 ± 0.03	1.71 ± 0.03	1.46 ± 0.02	1.41 ± 0.03	1.37 ± 0.02
OG_{S160} - HG_{S160}	1.00 ± 0.03	1.53 ± 0.04	1.91 ± 0.04	2.35 ± 0.14	2.75 ± 0.21
HG_{S160} - $NE2_{H237}$	1.84 ± 0.04	1.14 ± 0.03	1.04 ± 0.03	1.13 ± 0.03	1.85 ± 0.04
C_{PET} - O_{PET}	1.37 ± 0.02	1.41 ± 0.03	1.46 ± 0.03	1.77 ± 0.03	2.55 ± 0.03

	AI_b	TS 3	TI 2	TS 4	PC
OG_{S160} - C_{PET}	1.37 ± 0.02	1.42 ± 0.03	1.47 ± 0.03	1.58 ± 0.03	2.52 ± 0.03
O_{Water} - C_{PET}	2.45 ± 0.04	1.60 ± 0.03	1.45 ± 0.02	1.42 ± 0.03	1.37 ± 0.02
H_{Water} - $NE2_{H237}$	1.77 ± 0.04	1.32 ± 0.03	1.04 ± 0.03	1.15 ± 0.03	1.74 ± 0.04
O_{Water} - H_{Water}	1.01 ± 0.03	1.22 ± 0.03	1.86 ± 0.04	2.35 ± 0.16	3.09 ± 0.34
H_{Water} - OG_{S160}	2.68 ± 0.24	2.31 ± 0.11	2.10 ± 0.16	1.47 ± 0.04	1.02 ± 0.03

Table S2. Averaged distances (Å) between key atoms during 100ps AM1/MM MD simulation for different structures along the reaction mechanism explored for the PETase:$MHET_3$ model.

	RC	TS 1	TI 1	TS 2	AI_a
OG_{S160} - C_{PET}	3.05 ± 0.04	1.71 ± 0.03	1.46 ± 0.02	1.40 ± 0.02	1.37 ± 0.02
OG_{S160} - HG_{S160}	1.01 ± 0.03	1.15 ± 0.03	1.79 ± 0.04	2.35 ± 0.14	2.80 ± 0.20
HG_{S160} - $NE2_{H237}$	1.81 ± 0.04	1.43 ± 0.03	1.06 ± 0.03	1.17 ± 0.03	1.73 ± 0.04
C_{PET} - O_{PET}	1.37 ± 0.02	1.40 ± 0.03	1.46 ± 0.03	1.81 ± 0.03	2.54 ± 0.04

	AI_b	TS 3	TI 2	TS 4	PC
OG_{S160} - C_{PET}	1.37 ± 0.02	1.41 ± 0.03	1.47 ± 0.03	1.52 ± 0.02	2.60 ± 0.03
O_{Water} - C_{PET}	2.32 ± 0.03	1.68 ± 0.03	1.45 ± 0.02	1.43 ± 0.03	1.36 ± 0.02
H_{Water} - $NE2_{H237}$	1.68 ± 0.04	1.25 ± 0.03	1.02 ± 0.03	1.11 ± 0.03	1.72 ± 0.04
O_{Water} - H_{Water}	1.03 ± 0.03	1.26 ± 0.03	1.92 ± 0.04	2.27 ± 0.14	2.65 ± 0.22
H_{Water} - OG_{S160}	2.55 ± 0.17	2.34 ± 0.12	2.12 ± 0.17	1.54 ± 0.04	1.02 ± 0.03

Table S3. Averaged distances (Å) between key atoms during 100ps AM1/MM MD simulation for different structures along the reaction mechanism explored for the LCC-ICCG:$MHET_2$ model.

	RC	TS 1	TI 1	TS 2	AI_a
OG_{S165} - C_{PET}	3.20 ± 0.04	1.84 ± 0.03	1.45 ± 0.02	1.40 ± 0.02	1.39 ± 0.02
OG_{S165} - HG_{S165}	1.01 ± 0.03	1.22 ± 0.03	1.88 ± 0.04	2.43 ± 0.16	2.39 ± 0.14
HG_{S165} - $NE2_{H242}$	1.78 ± 0.04	1.31 ± 0.03	1.03 ± 0.03	1.18 ± 0.03	1.68 ± 0.04
C_{PET} - O_{PET}	1.37 ± 0.02	1.39 ± 0.02	1.47 ± 0.03	1.86 ± 0.03	1.93 ± 0.04

	AI_b	TS 3	TI 2	TS 4	PC
OG_{S165} - C_{PET}	1.37 ± 0.02	1.41 ± 0.03	1.47 ± 0.03	1.59 ± 0.03	2.83 ± 0.04
O_{Water} - C_{PET}	2.61 ± 0.04	1.69 ± 0.03	1.43 ± 0.02	1.41 ± 0.03	1.37 ± 0.02
H_{Water} - $NE2_{H242}$	1.76 ± 0.04	1.26 ± 0.03	1.01 ± 0.03	1.23 ± 0.03	1.73 ± 0.04
O_{Water} - H_{Water}	1.01 ± 0.02	1.24 ± 0.03	2.37 ± 0.05	2.39 ± 0.16	2.68 ± 0.31
H_{Water} - OG_{S165}	2.76 ± 0.21	2.33 ± 0.12	1.95 ± 0.11	1.31 ± 0.03	1.03 ± 0.03

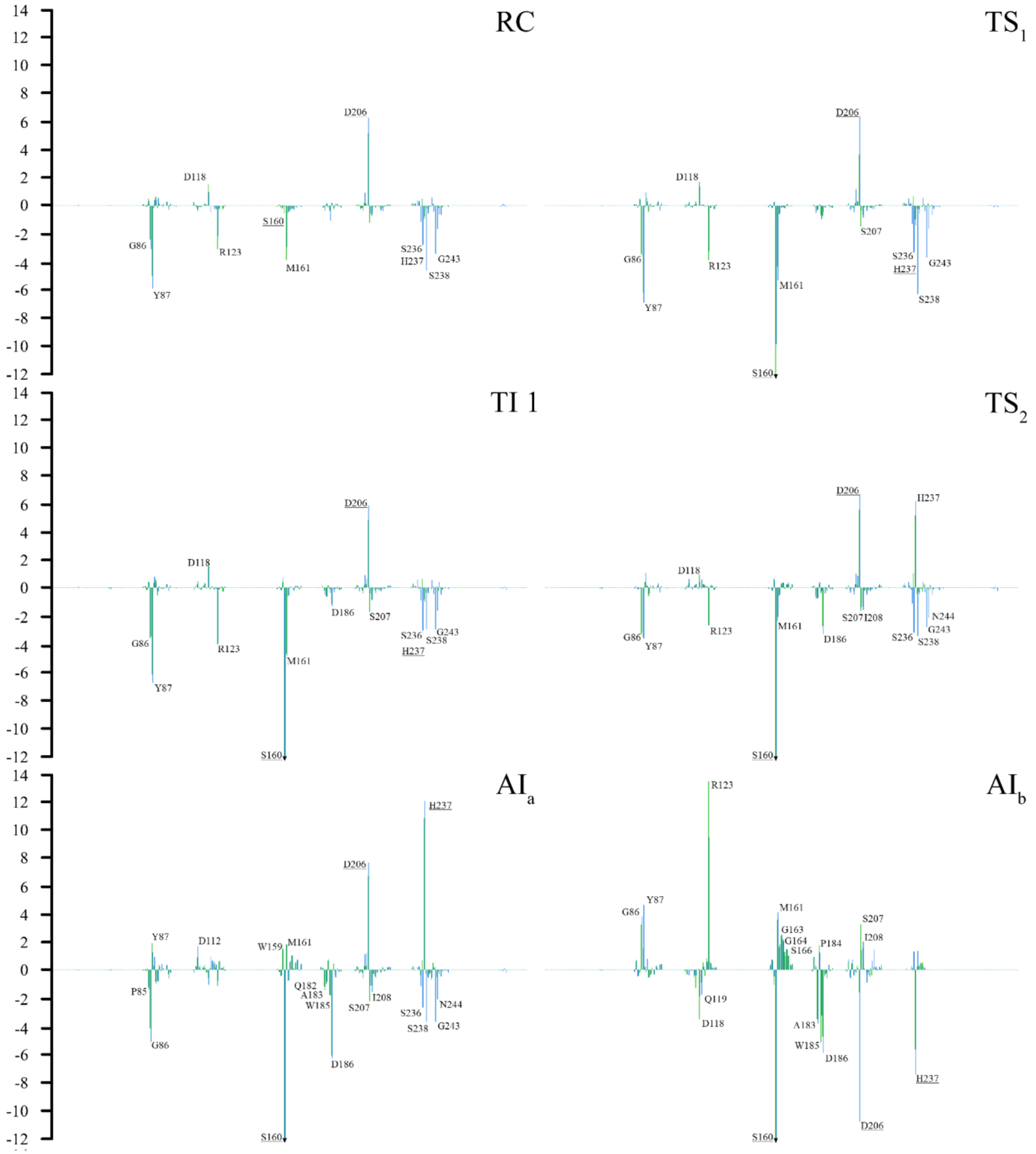

Figure S9. Electrostatic interaction energies (kcal·mol^{-1}) by residue, between PETase and the substrate: $MHET_2$ (green) and $MHET_3$ (blue). Only main contributor residues are displayed, being the catalytic triad underlined.

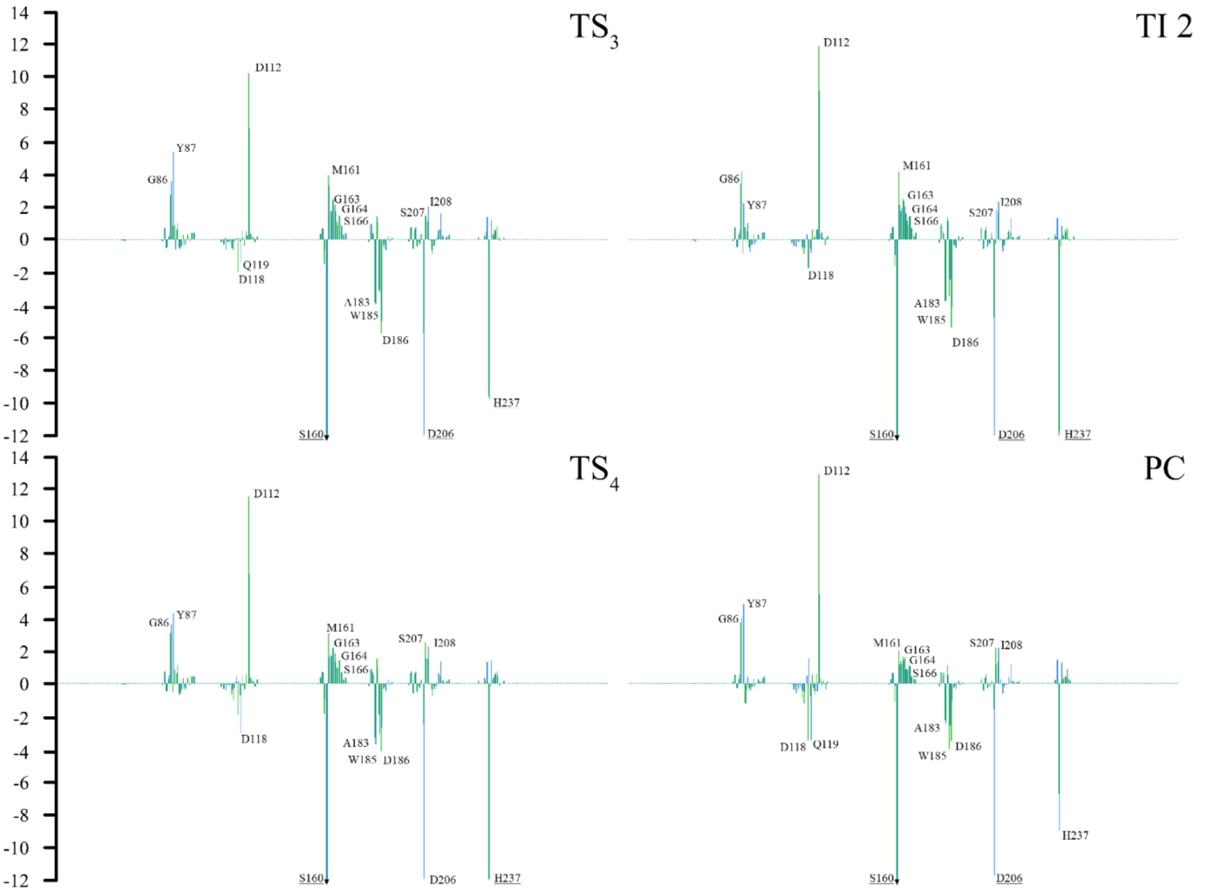

Figure S9 – Continuation. Electrostatic interaction energies (kcal·mol^{-1}) with the substrate for all the residues in the intermediates and transition structures of PETase with $MHET_2$ (green) and $MHET_3$ (blue). Only main contributor residues are displayed, being the catalytic triad underlined.

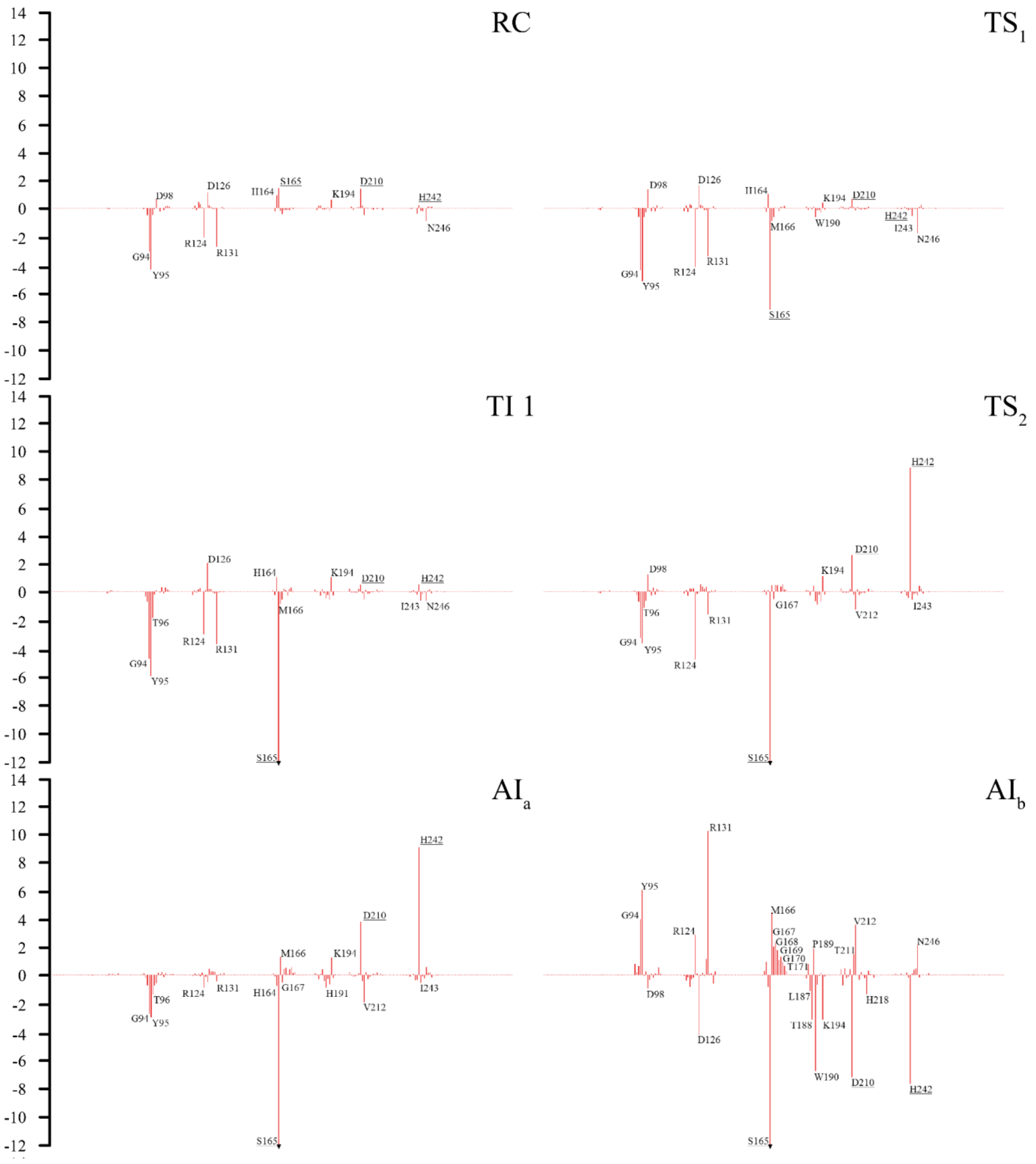

Figure S10. Electrostatic interaction energies (kcal·mol^{-1}) with the substrate for all the residues in the intermediates and transition structures of LCC-ICCG with $MHET_2$ (red). Only main contributor residues are displayed, being the catalytic triad underlined.

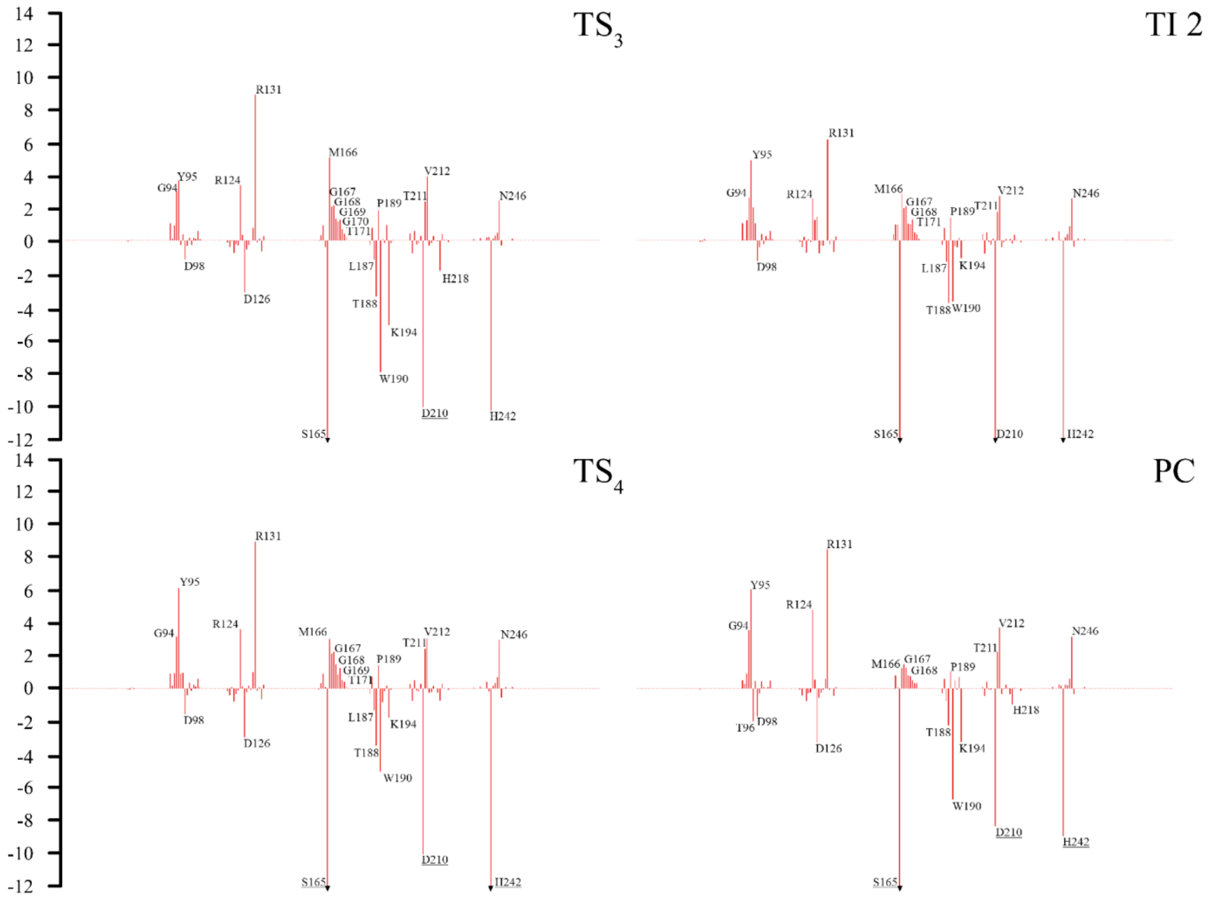

Figure S10 – Continuation. Electrostatic interaction energies (kcal·mol^{-1}) with the substrate for all the residues in the intermediates and transition structures of LCC-ICCG with $MHET_2$ (red). Only main contributor residues are displayed, being the catalytic triad underlined.

Table S4 – Cartesian coordinates (Å) of the atoms included in the QM region of the the PETase:$MHET_2$ system localized as transition states at M06-2X/6-31+G(d,p)//MM level.

TS1 (-539.2 cm^{-1})				TS2 (-539.8 cm^{-1})				TS3 (-431.8 cm^{-1})				TS4 (-420.4 cm^{-1})			
C	47.858	47.796	16.635	C	47.857	47.858	16.491	C	47.721	47.752	15.982	C	48.117	47.763	16.370
H	47.066	48.514	16.886	H	47.105	48.518	16.923	H	46.884	48.394	16.272	H	47.265	48.350	16.724
H	48.047	47.218	17.558	H	48.018	47.026	17.184	H	47.799	46.973	16.755	H	48.359	47.022	17.141
O	47.388	46.902	15.647	O	47.424	47.374	15.231	O	47.455	47.145	14.717	O	47.733	47.068	15.182
H	47.697	45.600	16.218	H	46.844	45.308	16.605	H	42.294	42.032	8.863	H	45.209	41.742	8.265
C	52.251	39.273	17.209	C	52.219	39.313	17.147	C	52.062	38.522	15.803	C	52.705	38.170	16.133
H	53.328	39.314	17.024	H	53.280	39.396	16.896	H	52.855	39.052	15.287	H	53.606	38.629	15.730
H	52.117	39.381	18.286	H	52.153	39.435	18.231	H	52.291	38.498	16.869	H	52.901	37.931	17.182
C	51.611	40.565	16.648	C	51.480	40.564	16.595	C	50.698	39.172	15.640	C	51.523	39.134	16.075
O	50.668	40.499	15.815	O	50.476	40.405	15.847	O	49.785	38.563	16.306	O	50.379	38.607	16.396
O	52.061	41.644	17.120	O	51.905	41.685	16.977	O	50.461	40.146	14.900	O	51.627	40.320	15.723

C	48.693	41.333	18.388	C	48.651	41.263	18.421	C	48.526	41.176	17.434	C	48.631	41.421	17.500
H	47.666	41.105	18.686	H	47.659	40.932	18.743	H	47.645	40.772	17.934	H	47.624	41.285	17.899
H	48.947	40.631	17.583	H	48.949	40.608	17.592	H	48.764	40.474	16.638	H	48.811	40.602	16.807
C	48.688	42.720	17.742	C	48.487	42.646	17.830	C	48.153	42.498	16.846	C	48.697	42.724	16.750
N	49.811	43.254	17.134	N	49.473	43.313	17.125	N	49.088	43.421	16.395	N	49.833	43.068	16.037
H	50.735	42.760	17.080	H	50.419	42.917	16.969	H	50.087	43.293	16.430	H	50.650	42.464	15.949
C	49.481	44.403	16.531	C	48.953	44.435	16.597	C	48.456	44.480	15.861	C	49.645	44.267	15.460
H	50.160	45.037	15.980	H	49.501	45.145	15.994	H	48.922	45.353	15.425	H	50.378	44.789	14.865
N	48.190	44.659	16.708	N	47.669	44.532	16.929	N	47.150	44.278	15.959	N	48.435	44.708	15.753
C	47.678	43.619	17.466	C	47.370	43.432	17.701	C	46.933	43.055	16.563	C	47.825	43.762	16.552
H	46.634	43.599	17.738	H	46.376	43.295	18.089	H	45.947	42.671	16.759	H	46.814	43.909	16.900
C	46.010	46.241	11.591	C	46.010	46.217	11.536	C	46.424	46.640	10.550	C	46.992	45.942	11.309
C	44.849	45.477	11.442	C	44.810	45.508	11.490	C	45.568	45.565	10.295	C	45.753	45.424	10.933
C	43.942	45.373	12.497	C	43.973	45.458	12.611	C	44.938	44.936	11.368	C	44.659	45.577	11.787
C	44.211	46.007	13.703	C	44.354	46.103	13.783	C	45.175	45.378	12.668	C	44.823	46.161	13.039
C	45.410	46.709	13.890	C	45.596	46.735	13.861	C	45.959	46.503	12.940	C	46.071	46.638	13.449
C	46.294	46.841	12.821	C	46.410	46.809	12.731	C	46.596	47.113	11.852	C	47.141	46.562	12.551
C	44.610	44.650	10.226	C	44.520	44.674	10.292	C	45.353	45.099	8.880	C	45.626	44.646	9.663
O	44.875	45.276	9.076	O	44.813	45.285	9.138	O	44.238	44.351	8.792	O	44.386	44.720	9.157
O	44.223	43.496	10.286	O	44.113	43.529	10.362	O	46.071	45.373	7.954	O	46.530	44.014	9.156
C	45.627	47.371	15.243	C	46.033	47.431	15.124	C	46.045	47.123	14.356	C	46.177	47.250	14.853
O	44.988	46.560	16.165	O	45.738	46.145	16.375	O	45.416	48.211	14.593	O	45.820	48.473	15.024
O	45.585	48.619	15.364	O	45.469	48.493	15.479	H	46.938	47.108	9.713	H	47.834	45.847	10.628
C	44.882	46.968	17.505	C	45.281	46.664	17.593	H	44.298	44.078	11.181	H	43.683	45.225	11.468
C	43.691	46.235	18.087	C	43.876	46.197	17.924	H	44.761	44.817	13.496	H	43.964	46.218	13.704
H	42.772	46.561	17.596	H	43.143	46.620	17.233	H	47.234	47.974	12.027	H	48.106	46.963	12.842
H	46.717	46.317	10.769	H	46.657	46.255	10.664	C	43.785	43.879	7.511	C	44.125	44.078	7.903
H	43.024	44.803	12.379	H	43.026	44.925	12.566	C	43.718	42.357	7.509	C	43.513	42.705	8.097
H	43.494	45.936	14.511	H	43.710	46.085	14.655	O	43.248	41.833	8.746	O	44.388	41.802	8.768
H	47.223	47.384	12.965	H	47.371	47.309	12.792	H	42.794	44.313	7.359	H	43.406	44.719	7.387
H	45.460	37.864	13.981	H	45.639	37.863	14.001	H	44.448	44.233	6.718	H	45.046	44.031	7.315
H	45.801	46.711	18.049	H	45.973	46.379	18.404	H	43.078	42.044	6.673	H	42.608	42.761	8.707
H	44.706	48.049	17.568	H	45.243	47.764	17.546	H	44.707	41.921	7.358	H	43.234	42.315	7.109
H	43.636	46.381	19.167	H	43.617	46.453	18.955	O	45.472	46.016	15.291	O	45.584	46.341	15.766
C	43.469	42.374	15.258	C	43.527	42.325	15.213	H	46.381	45.051	15.674	H	48.025	45.779	15.428
C	43.737	42.895	16.526	C	43.706	42.829	16.502	H	44.943	46.532	15.919	H	45.109	46.899	16.396
C	44.451	42.140	17.461	C	44.347	42.061	17.477	H	49.318	41.163	18.045	H	49.316	41.296	18.217
C	44.923	40.881	17.114	C	44.866	40.817	17.146	H	52.015	37.566	15.513	H	52.505	37.294	15.694
C	44.682	40.372	15.835	C	44.723	40.328	15.844	H	48.572	48.278	15.979	H	48.909	48.362	16.250
C	43.938	41.111	14.910	C	44.025	41.069	14.885								
C	43.270	44.284	16.825	C	43.276	44.234	16.766								
O	43.882	44.823	17.886	O	43.868	44.760	17.840								
O	42.436	44.865	16.166	O	42.502	44.843	16.056								
C	45.298	39.062	15.462	C	45.394	39.045	15.482								

O	45.003	38.686	14.229	O	45.156	38.672	14.236
O	46.036	38.424	16.199	O	46.125	38.423	16.238
C	44.921	44.453	7.882	C	44.906	44.454	7.953
C	44.600	45.297	6.677	C	44.619	45.296	6.737
O	43.208	45.574	6.524	O	43.232	45.575	6.551
H	42.908	42.972	14.547	H	43.022	42.934	14.471
H	44.617	42.539	18.457	H	44.429	42.442	18.490
H	45.480	40.282	17.827	H	45.387	40.219	17.887
H	43.757	40.720	13.913	H	43.912	40.689	13.875
H	45.925	44.014	7.808	H	45.918	44.029	7.912
H	44.186	43.650	7.957	H	44.176	43.646	8.008
H	45.172	46.235	6.711	H	45.193	46.234	6.789
H	44.906	44.729	5.793	H	44.950	44.730	5.861
H	42.760	45.676	7.384	H	42.767	45.672	7.402
H	49.301	41.134	19.157	H	49.292	41.108	19.173
H	51.878	38.385	16.941	H	51.861	38.410	16.908
H	48.690	48.319	16.452	H	48.714	48.362	16.386
